# Efficient quenching sheds light on early stages of gold nanoparticle formation†

**DOI:** 10.1039/d3ra02195e

**Published:** 2023-06-14

**Authors:** Markus Biegel, Tobias Schikarski, Paola Cardenas Lopez, Lukas Gromotka, Christian Lübbert, Andreas Völkl, Cornelia Damm, Johannes Walter, Wolfgang Peukert

**Affiliations:** a Institute of Particle Technology (LFG), Friedrich-Alexander-Universität Erlangen-Nürnberg (FAU) Cauerstrasse 4 91058 Erlangen Germany wolfgang.peukert@fau.de; b Interdisciplinary Center for Functional Particles Systems (FPS), Friedrich-Alexander-Universität Erlangen-Nürnberg (FAU) Haberstraße 9a 91058 Erlangen Germany

## Abstract

The formation mechanism of plasmonic gold nanoparticles (Au NPs) by fast NaBH_4_ induced reduction of the precursors is still under debate. In this work we introduce a simple method to access intermediate species of Au NPs by quenching the solid formation process at desired time periods. In this way, we take advantage of the covalent binding of glutathione on Au NPs to stop their growth. By applying a plethora of precise particle characterization techniques, we shed new light on the early stages of particle formation. The results of *in situ* UV/vis measurements, *ex situ* sedimentation coefficient analysis by analytical ultracentrifugation, size exclusion high performance liquid chromatography, electrospray ionization mass spectrometry supported by mobility classification and scanning transmission electron microscopy suggest an initial rapid formation of small non-plasmonic Au clusters with Au_10_ as the main species followed by their growth to plasmonic Au NPs by agglomeration. The fast reduction of gold salts by NaBH_4_ depends on mixing which is hard to control during the scale-up of batch processes. Thus, we transferred the Au NP synthesis to a continuous flow process with improved mixing. We observed that the mean volume particle sizes and the width of the particle size distribution decrease with increasing flow rate and thus higher energy input. Mixing- and reaction-controlled regimes are identified.

## Abstract 2

Quenching by glutathione enables visualization of intermediate stages of the gold nanoparticle formation. A T-mixer allows precise control of the particle size distribution *via* the Reynolds number Re.

## Graphical abstract

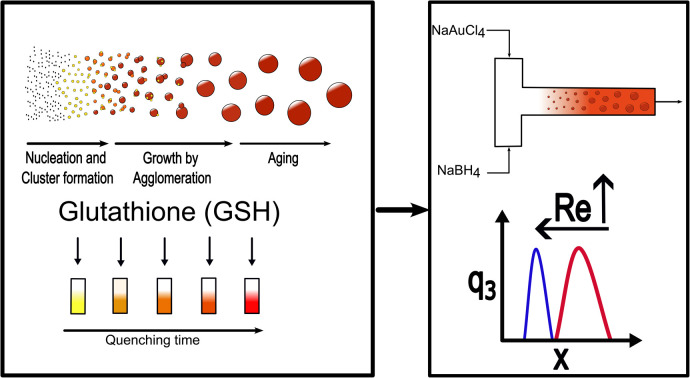

## Introduction

Gold nanoparticles (Au NPs) can be used for a variety of applications including sensors, medicine and catalysis.^1–3^ Process control is key to tailor the optical product properties of plasmonic NPs by modulation of their size and shape. In order to control particle formation in the best possible way, the determining driving forces must be known. For the production of Au NPs < 5 nm, a high nucleation rate by fast reduction of the Au precursor is required. This results in a mixing controlled particle formation process. Therefore, reliable synthesis processes to control the particle size distribution (PSD) are necessary for the design of their characteristic optical and electronic properties, known as localized surface plasmon resonance (LSPR). In contrast to batch processes, mixing in continuous flow processes can be controlled by proper adjustment of the fluid flow and local energy dissipation.^4–8^ In comparison with batch synthesis of Au NPs much less papers deal with continuous flow synthesis. The continuous processes used so far to synthesize Au NPs are rather complex.^9,10^ Often, micromixers are designed as microchips with diameters well below 100 μm largely limiting scale-up. Multiple inlets into the mixer and changes in diameter of the flow channel result in complex flow patterns with the tendency for clogging.^11,12^ Microfluidic coaxial flow reactors (CFR) are used alone or in combination with coiled flow inverters (CFI) or split and recombine (SAR) mixers.^13,14^ In CFRs the flow through the outer and the inner tube creates an interface between the reactant streams, where the reaction takes place. The CFI is used for adapting the residence time. By splitting and recombining the streams by thicker and thinner interdigitated lamellae, the SAR is improving the mixing by reducing the distance of diffusion.^13,14^ A further reactor type used for metal NP synthesis are segmented flow reactors, where the reaction phase is injected into the channel alongside an immiscible fluid dividing the reacting agents into nearly-identical droplets.^15,16^ In all these cases, the detailed modelling of the fluid flow in small and complex channels is challenging and may be further complicated by the impact of the channel surface. This hampers a better understanding of the mechanism of mixing controlled Au NP formation and limits scale-up.

The formation mechanism of small Au NPs ≤5 nm is discussed in many publications^1,17–19^ but is not yet fully understood. Generally, Au NPs are formed by a multistep process consisting of the formation of Au atoms by reduction of the precursor, nucleation due to supersaturation of Au atoms and growth of the nuclei. A measurable amount of free Au atoms was detected by Abecassis *et al.* using X-ray absorption near-edge structure spectroscopy (XANES) and small angle X-ray scattering (SAXS).^20^ According to the authors, the rate at which Au atoms appear in the bulk solution, and thus the supersaturation, is the key point that controls the balance between nucleation and growth.^20^ Based on SAXS and small angle neutron scattering (SANS) measurements, Polte *et al.*^18,21^ postulated that, after rapid reduction of Au(iii) to Au(0), molecular Au clusters are formed by nucleation, which grow in the following by agglomeration to plasmonic Au NPs. Au nanoclusters as intermediates in the Au NP formation process were also detected by *in situ* transmission electron microscopy (TEM) studies using a liquid cell.^22^ The detected amorphous Au clusters are the result of nucleation of Au atoms in Au atom rich solution zones, which are formed by spinodal decomposition. The amorphous Au clusters were found to crystallize in the next step.^22^

The classical mechanism according to LaMer^23,24^ states an initial homogeneous nucleation burst from a supersaturated solution merging into a spontaneous diffusive, agglomerative growth of monodispersed hydrosols. Chen *et al.* used SAXS, wide angle X-ray scattering (WAXS) and UV/vis data and described the formation of Au NPs due to Au-salt reduction by an organoborate in toluene by the LaMer model.^25^ Shields *et al.* described sigmoidal Au nanocrystal growth kinetics by an aggregative nucleation step followed by growth due to coalescence of the aggregates.^26^ For the Au NP formation due to reduction of an Au-salt by triblock copolymers, a model based on aggregative nucleation and growth by Ostwald ripening was proposed.^27^ This approach uses a combination of the Kolmogorov–Johnson–Mehl–Avrami model for phase transition with Ostwald ripening. In a number of review papers the Finke group provides evidence that the LaMer model does not hold for slow particle formation processes.^24,28^ Instead, they described the formation kinetics of Au NPs quantitatively based on their Watzky and Finke model,^17^ which proposes a general mechanism consisting of two pseudo elementary reactions: a slow, continuous nucleation followed by a subsequent rapid autocatalytic surface growth. This model was originally derived to describe the catalytic hydrogenation activity of Ir(0) nanoclusters. Later, this approach was transferred to several other examples including the seed-mediated synthesis of single-crystalline Au NPs^29^ and the formation of spherical Au NPs by using the weak reducing agents chitosan^30^ or cyclodextrin.^31^ The reduction-crystallization model proposed by Zhou *et al.* emerged in line with the Watzky and Finke model.^32^ Despite the considerable progress in understanding and modelling the formation of small nanoparticles, many open questions remain in particular for the understanding of early reaction steps and the role of intermediate species.

Here we demonstrate a facile way to quench the rapid synthesis of Au NPs at defined short times by addition of an efficient stabilizer. Thiol groups containing ligands like glutathione (GSH)^33^ or biological macromolecules such as lysozyme^34^ or pepsin^35^ are known to be efficient stabilizing agents for ultra-small Au clusters. For our mechanistic investigations of Au NP formation the small, fast diffusing molecule GSH is more suitable. Analysis of the characteristic LSPR of the quenched intermediate species by UV/vis spectroscopy in combination with precise *ex situ* methods like size-exclusion high performance liquid chromatography (HPLC-SEC),^36^ electrospray ionization mass spectrometry supported by mobility classification (ESI-DMA-MS)^37–40^ and analytical ultracentrifugation (AUC)^41,42^ reveals the formation of non-plasmonic Au clusters with Au_10_ as main species on early stages and their transformation to plasmonic Au NPs.^43–45^ In particular, AUC and HPLC-SEC are highly accurate, reliable and reproducible methods to measure the size distribution of small Au NPs in suspension with excellent statistics.

Our methods provide quantitative data on rapid growth at short time scales and slow growth of Au NPs on a longer time scale. In addition, we apply continuous flow synthesis of Au NP <5 nm in a simple T-mixer to vary systematically the flow rate and thus the Reynolds number (Re) to study the mixing behaviour of the educt solutions and its effect on the PSD of the formed Au NPs.

## Material and methods

### Chemicals

All chemicals were used for Au NP synthesis as received from the manufacturers. The syntheses were carried out in aqueous solutions at 20 °C, with Millipore water (18 MΩ cm) as solvent. Sodium tetrachloroaurate dihydrate (NaAuCl_4_·2H_2_O, 99%, Sigma-Aldrich, 397.8 g mol^−1^) was used as precursor. For a fast reduction of the Au salt, the strong reduction agent sodium borohydride (NaBH_4_ > 97%, Carl Roth, 37.83 g mol^−1^) was used. To quench the synthesis at a defined time, an aqueous solution of the stabilizing agent l-glutathione (GSH, ≥98.0%, Sigma-Aldrich, 307.32 g mol^−1^) was added. Furthermore, 10 mM sodium hydroxide (NaOH 99.8%) solution (Carl Roth, 40.01 g mol^−1^) and boric acid (≥99.5%, Sigma-Aldrich, 61.83 g mol^−1^) were used to adjust the pH in all syntheses. Sodium dodecylsulfate (SDS ≥ 99%, Sigma-Aldrich) and ammonium acetate (“BioUltra” ≥ 99%, Sigma-Aldrich) were used to tailor the interactions between the Au NPs and the stationary phase material in the HPLC-SEC experiments.

### Au NP batch synthesis

To prepare the Au NPs in batch, a snap cap tube with a volume of 10 mL was used. The reaction vessel and the magnetic stirring bar used were cleaned with aqua regia and rinsed with Millipore water prior to use. Two solutions were prepared, the precursor and the reduction agent solution. The precursor solution consisted of 19.2 mM boric acid as buffer to adjust the pH of the reaction mixture to 7.4 and of 0.2 mM NaAuCl_4_ in Millipore water. The reduction agent solution consisted of 0.6 mM NaBH_4_ stabilized by 0.54 mM NaOH. As both solutions are mixed in volume ratio 1 : 1, the concentrations in the reactor are 9.6 mM for boric acid, 0.1 mM for NaAuCl_4_ and 0.3 mM for NaBH_4_. 5 mL of the precursor solution was transferred to the reaction vial and stirred vigorously. Subsequently, 5 mL of the reduction agent solution was added rapidly, which led to an immediate color change from transparent to orange-brown. Within the next 5 min, the color of the reaction mixture turned to red, while stirring continued during this time.

Due to the fast reactions, it was necessary to quench the synthesis on a short time scale, especially to analyse intermediate species. The Au NP samples were quenched with GSH and are referred to as GSH-Au NPs. 0.27 mL of a 15 mM GSH solution was added rapidly to quench the Au NP batch synthesis. For the sample quenched at 0 s, GSH was added to the precursor solution prior to the reduction agent solution.

### Continuous flow synthesis

**Fig. 1:**
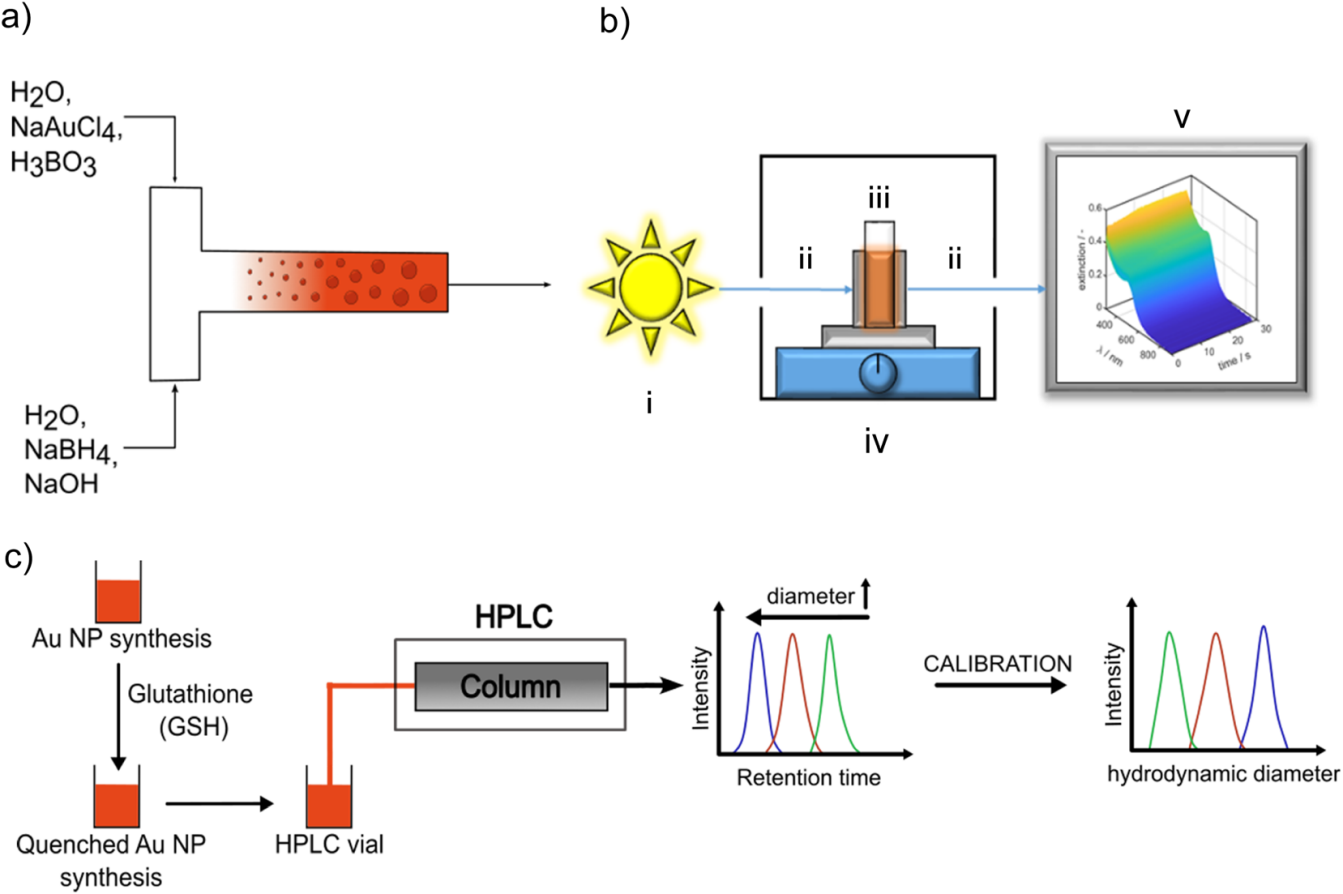
(a) Scheme of continuous flow synthesis; (b) scheme of the *in situ* fiber UV-vis spectrometer, (i) light source, (ii) fiber optics, (iii) housing, (iv) cuvette holder on a magnetic stirrer, (v) PC for data recording and processing, (c) scheme of quenching the Au NP growth by adding GSH and measurement of the PSDs of the quenched samples by HPLC-SEC by using a calibration curve for assigning the measured retention time distribution to a PSD.

For continuous flow synthesis of Au NPs, a syringe pump (KDS, Legato 200) with two syringes (Mono-Ject) having a maximum filling volume of 140 mL each, silicone tubing with an inner diameter of 3 mm and a length of 30 cm, and a T-mixer (acrylonitrile–butadiene–styrene copolymer ABS) with an inner diameter of 1 mm at the inlet and outlet were used. A schematic illustration of the setup is shown in Fig. 1a. The syringes were rinsed at least three times with Millipore water before and after each synthesis. The mixer and silicone tubing were cleaned three times with Millipore water.

Syringe 1 was filled with the precursor solution consisting of 0.2 mM NaAuCl_4_ in 19.2 mM boric acid buffer solution. Syringe 2 contained the reducing agent solution which included 0.6 mM NaBH_4_ and 0.54 mM NaOH in aqueous solution. The concentrations in the reaction system were 9.6 mM for boric acid, 0.1 mM for NaAuCl_4_ and 0.3 mM for NaBH_4_ as in the T-mixer both solutions are mixed in volume ratio 1 : 1. Each syringe was filled with 120 mL of the respective solution, resulting in a total volume of 240 mL. The flow rate was continuously adjustable in a range from 3.1 pL min^−1^ to 216 mL min^−1^, which translated into a range of the Reynolds number Re, which is defined as1
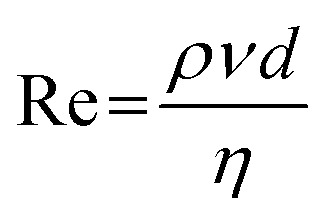
with *ρ*, *η* and *v* are the density, the dynamic viscosity and the velocity of the fluid, respectively, *d* is the characteristic length, which is in our case the inner diameter of the T-mixer inlet. Re was varied experimentally between 30 and 3000 at a temperature of 20 °C.

### UV/vis spectroscopy (UV/vis)

The LSPR of the Au NPs is detectable by UV/vis spectroscopy. The typical extinction spectrum of non-agglomerated spherical plasmonic Au NP exhibits a valley at 450 nm and a single LSPR peak between 500 nm and 520 nm depending on Au NP size. Au clusters do not exhibit a LSPR peak.^33,46,47^ By tracking the UV/vis spectra during the synthesis, the temporal evolution of the optical properties and hints on intermediate species are accessible. A Lambda 35 UV/vis spectrometer (PerkinElmer) and disposable cuvettes with a path length of 1 cm were used for benchtop measurements in the wavelength range of 250–1100 nm. A scheme of the *in situ* UV/vis spectrometer setup can be seen in Fig. 1b. It consists of a light source including a tungsten and deuterium lamp (i) in Fig. 1b) (Avantes Avalight-DH-S-BAL) which is able to cover a wavelength range from 250–2500 nm. For our measurements the wavelength range is set to 300–900 nm with a minimum time resolution of 1.05 ms. The light source is connected with a cuvette holder for a polystyrene (PS) disposable cuvette, (iv) in Fig. 1b, (2.5 mL Makro, optical path 1 cm, BRAND GmbH) *via* a fiber optics, (ii) in Fig. 1b (Avantes FC-UVIR100-2). In the cuvette holder also a stirring plate for a magnetic stirrer is implemented to ensure a good mixing behaviour. This holder is then connected with a second fiber optics, (ii) in Fig. 1b, (Avantes FC-UVIR100-2) to the detector (Avantes AvaSpec-2048L). Recorded time-dependent UV/vis spectra were saved by Avasoft (Version: 8.10) and processed with MATLAB.

### Analytical ultracentrifugation (AUC)

PSDs of the obtained Au NPs were measured in suspension as a function of the synthesis parameters by sedimentation velocity (SV) AUC experiments. The data was acquired with a commercial analytical ultracentrifuge, type Optima AUC, from Beckman Coulter (Brea CA, USA). In order to avoid ripening of the boric acid-stabilized Au NPs, the samples were stored at 5 °C immediately after synthesis until the SV-AUC measurements were performed. All samples were measured without further purification and dilution in both titanium and 3D printed centrepieces^48^ with an optical path length of 12 mm. SV-AUC experiments on boric acid-stabilized Au NPs were performed at 5 °C and GSH-Au NPs were measured at 20 °C. Sedimentation data was acquired with at least 300 scans, a radial step of 50 μm, a rotor revolution of 15 000 rpm (relative centrifugal force RCF: 16 380*g*) and a detection wavelength of 350 nm. In the case of GSH-Au NPs, the rotor speed was 25 000 rpm (RCF: 45 500*g*) and the wavelength was 250 nm.

The acquired SV data for the Au NPs was analyzed with the ls-g*(s) model in the software Sedfit (version 16-1c).^49^ The data was fitted with the second derivative regularization using a confidence level of 0.90 and resolution of 150 grid points.

To obtain the GSH shell thickness, a sample consisting primarly of Au_10_(GSH)_10_ clusters was measured in an SV experiment at 20 °C and 50 000 rpm (RCF: 182 000*g*). The detection wavelength was 300 nm. The retrieved data was analysed with the *c*(*s*) continuous size distribution model in Sedfit. The data was fitted with the maximum entropy regularization using a confidence level of 0.683 and a resolution of 150 grid points. Further details can be found in the ESI (Fig. S1†).

### Size exclusion high performance liquid chromatography (HPLC-SEC)

PSDs of Au NPs quenched with GSH after defined reaction times were characterized by HPLC-SEC. For the chromatographic experiments, an Ultimate 3000 UHPLC setup (Thermo Fisher Scientific Inc., Waltham, MA, USA) was used. The setup includes a solvent rack (SR-3000), a quaternary pump (LPG-3400SD), an autosampler (WPS-3000SL), a column thermostat (TCC-3000RS), a diode array detector (DAD-3000) and a fraction collector (Fraction collector F). For the separation, a Reprosil Saphir Si column (Altmann Analytik, Munich, Germany) with unmodified silica particles (10 μm particle size, 100 nm pore size) was used. The column has an inner diameter of 8 mm and a length of 300 mm. As mobile phase, an aqueous solution of 2 mM sodium dodecyl sulfate (SDS) and 8 mM ammonium acetate was used at a flow rate of 0.5 mL min^−1^. All HPLC-SEC experiments were performed at 25 °C and chromatograms were recorded using a detector wavelength of 520 nm. An universal calibration curve for assigning the measured retention times (or volumes) to a particle size was obtained using various citrate-stabilized Au NP dispersions with narrow PSDs (mean diameter: 5, 10, 20, 30, 40, 50, 60, 80 nm) purchased from nanoComposix and an additional gold nanocluster sample synthesized as described in literature.^46^ A scheme of HPLC-SEC analysis of quenched Au NP samples is shown in Fig. 1c. Further details on the calibration of HPLC-SEC can be found in the ESI (Fig. S2 and Table S1†) and in our previous paper.^36^

### Dynamic light scattering (DLS)

The PSDs of the Au NP dispersions for the construction of the HPLC-SEC calibration curve were analyzed by dynamic light scattering using a Zetasizer Nano-ZS (Malvern Panalytical Ltd, Malvern, United Kingdom) after diluting 100 μL of suspension in 2 mL mobile phase solution (2 mM SDS and 8 mM ammonium acetate in water). The volume mean NP sizes were measured at 25 °C and 173° backscattering angle. Each measurement was performed three times.

### Electrospray ionization mass spectrometry supported by mobility classification (ESI-DMA-MS)

GSH-Au NP samples, quenched at 0 s, 2 s and 5 s were analyzed by ESI-DMA-MS. As excess GSH and boric acid would disturb the detection of the Au clusters, the samples were purified prior to ESI-DMA-MS analysis. The dried samples were dispersed in a washing solution consisting of 20 mL methanol, 25 mL 2-propanol and 5 mL of 20 mM aqueous formic acid by bath sonication for 15 min and then four washing steps were performed. In each washing step the samples were centrifuged at 12 000 rpm (RCF: 12 500*g*) for two minutes using a “Centrifuge 5418” (Eppendorf, Hamburg, Germany). The supernatant was removed and the sediment was dispersed in the washing solution by bath sonication for 5 min. After the last centrifugation step, the sediment was dispersed in 1 mL aqueous solution of 50 mM ammonium acetate and 10 mM ammonia. Hereby, more than 90% of soluble contaminations were removed. These dispersions were finally transferred to gas phase by electrospray ionization, classified by their mass/charge ratio in the electric field and analyzed by MS. The ESI-DMA-MS setup and technique are described in more details by Lübbert *et al.*^37,38^

## Results & discussion

### Batch synthesis – quenching particle growth by fast and efficient stabilization

NaBH_4_ reduces Au(iii) ions to elementary gold^50,51^ which is described by following overall reaction:

2 Au^3+^ + BH_4_^−^ + 4H_2_O → Au^0^ + [B(OH)_4_]^−^ + 3H^+^ + 0.5H_2_

**Fig. 2:**
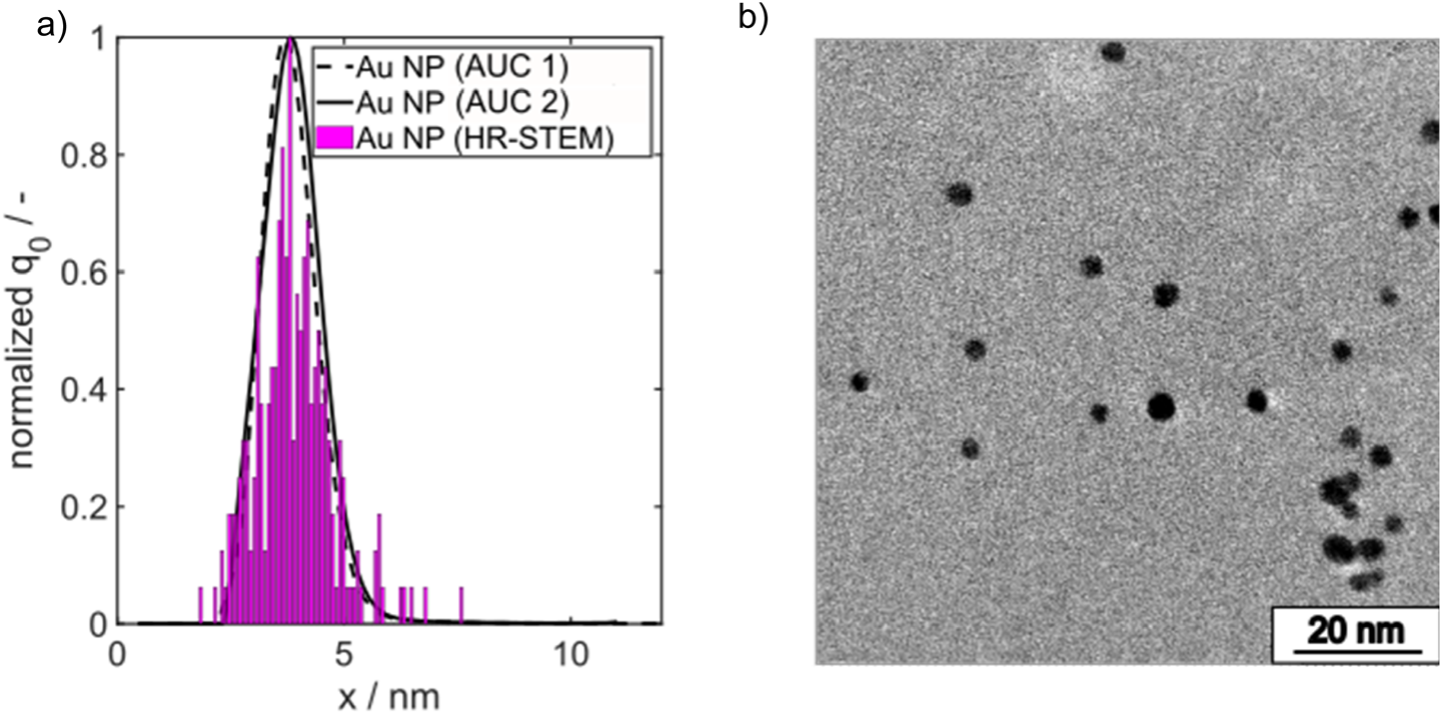
(a) Comparison of PSDs measured by AUC and HR-STEM from batch-synthesized Au NPs. (b) Representative HR-STEM micrograph of spherical batch synthesized Au NPs.

A minimum molar ratio between Au(iii) and borohydride ions of 1 : 1 is required for full conversion of Au(iii) ions. Note that we kept the Au salt concentration always fixed at 0.1 mM in the final reaction volume and the molar Au salt to NaBH_4_ concentration ratio was set to 1 : 3 to ensure complete conversion. The final PSD of the Au NPs was measured by high resolution scanning transmission electron microscopy (HR-STEM) and AUC. The number density core PSDs *q*_0_(*x*) measured by both methods agree very well as shown in Fig. 2a. The spherical morphology is depicted in Fig. 2b.

**Fig. 3:**
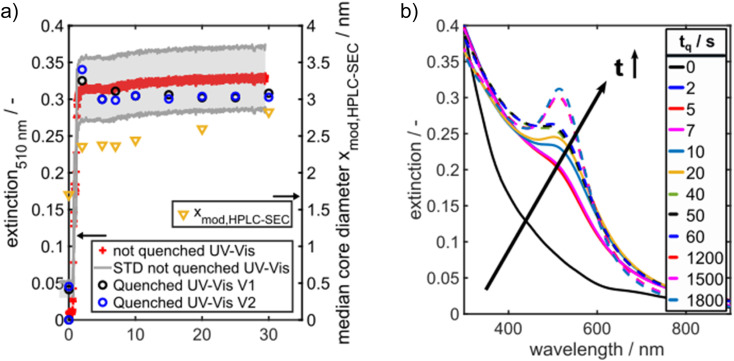
(a) Extinction at 510 nm over time. Different kinetic regimes are visible which start from a rapid nucleation and slow down to a much slower growth (left axis). The black and blue data points belong to two different quenching experiments “V1” and “V2”, performed under identical conditions to check the reliability of the quenching method. On the right axis, the median core diameter is shown as measured by HPLC-SEC (yellow data points). The particle number concentration decreases from 6.1 × 10^20^ m^−3^ in the beginning to 3.0 × 10^19^ m^−3^ at the end. (b) *In situ* UV/vis spectra as a function of quenching time *t*_q_. The spectrum at 0 s refers to a preliminary mixture of precursor solution and GSH before the reduction agent solution was added.

The Au NP formation due to Au salt reduction by NaBH_4_ is fast, which makes the detection of intermediate species challenging. To get first insights into the formation kinetics, we tracked the Au NP formation by *in situ* UV/vis spectroscopy. Recording of the UV/vis spectra was started two seconds before starting the Au NP formation. The time axis in Fig. 3a was shifted such that 0 s corresponds to the onset of the solid formation. A typical evolution of the extinction at the LSPR wavelength of 510 nm, denoted in the following as E510, can be seen in Fig. 3a. The detection wavelength of 510 nm was selected as it corresponds to the LSPR peak maximum of spherical Au NPs ≤5 nm. E510 increases rapidly within the first 2 s followed by a very slow further increase within 30 s (red crosses in Fig. 3a). The grey region in Fig. 3a shows the standard deviation of the extinction measurement. The measured temporal evolution of E510 hints to fast particle formation followed by a slow further particle growth process, but the relatively high standard deviation indicates that tracking of the Au NP formation dynamics by “simple” *in situ* UV/vis spectroscopy is hampered by scattered light due to H_2_ bubbles.

To get more information regarding intermediate species, we developed a facile method to freeze the Au NP formation process by adding GSH as a quencher at different time delays after starting the reaction. GSH is a well-known and highly efficient stabilizer of Au clusters and small Au NPs^46,52^ as it binds covalently to the surface of the Au NPs and therefore prevents their further growth. It should be noted, that GSH is able to reduce Au(iii) ions to Au(i) ions and forms Au(i) complexes. The strong reducing agent NaBH_4_ is added to the Au-salt at first and the GSH is added after a defined time delay. Therefore, the interference of GSH on the mechanism of Au NP formation is expected to be low as the Au(iii) ions are much more rapidly reduced by the NaBH_4_ than by GSH, whose reducing power is much weaker compared to NaBH_4_. Mixing of GSH to the suspension of growing Au NPs with subsequent diffusion and binding of GSH to the surface of the forming Au NPs requires a certain time. The sample quenched without time delay “*t*_q_ = 0 s” represents the state after the mixing time. In the following we will show that cluster and NP growth is quenched efficiently.

The evolution of the solid formation process is visualized by UV/vis spectra recorded for different quenching times *t*_q_, see Fig. 3b. The blue and black data points in Fig. 3a show E510 of the quenched samples from two independent quenching experiments (marked with “V1” and “V2” in Fig. 3b) over time. A comparison of the black and blue data points shows that the quenching experiments are well reproducible. Moreover, the extinction values of the quenched samples agree with the values of the not quenched samples (red crosses in Fig. 3a) within the experimental error which demonstrates the reliability of the quenching method.

The time-dependent UV/vis spectra reveal an initial, fast solid formation within the first 2 s. The spectrum obtained after the mixing time (*t*_q_ = 0 s) does not exhibit any LSPR peak at 500–520 nm (Fig. 3b). The typical peaks of Au_10_ clusters between 350 and 400 nm (ref. 53) are also not visible, but the extinction in this wavelength range is rather high, suggesting the presence of a mixture of different small Au species. ESI-DMA-MS investigations on the *t*_q_ = 0 s sample reveal a mixture of Au_9_–Au_12_ clusters with Au_10_ being the most frequently occurring species (see Fig. S3 in the ESI†). Moreover, the remarkable extinction at wavelengths >400 nm indicates the presence of Au species larger than Au_10_ which do not appear in the MS spectrum.^53^ The samples quenched at times until 7 s exhibit no clear LSPR peak, but a shoulder at around 500 nm, which suggests that the conversion of Au clusters to plasmonic Au NPs has already started and proceeds with time. After 10 s a LSPR peak is visible and becomes more prominent for longer reaction times (Fig. 3b). As the extinction data provide limited information on the particle size, the quenched GSH-Au NP samples were additionally analysed regarding their PSD with HPLC-SEC. HPLC-SEC tracks the hydrodynamic diameter of the Au NPs, which is the sum of the Au core diameter and twice of the GSH shell thickness. The reliability of HPLC-SEC for determination of PSDs was validated by AUC in our previous paper.^36^ The GSH shell thickness was obtained as 0.86 nm from the diffusion corrected sedimentation coefficient distribution of a Au_10_GSH_10_ cluster sample measured by AUC (see Fig. S1 in the ESI†). The temporal evolution of the median values of the core diameters of the Au NPs is similar to the temporal evolution of E510, see yellow triangles in comparison with the black and blue data points in Fig. 3a.

In the following, we present PSDs for samples taken at different quenching times and analysed *ex situ* by HPLC-SEC.^36^ The underlying calibration curve for assigning the measured retention time to a particle size is given in the ESI (Fig. S2 and Table S1†).

**Fig. 4:**
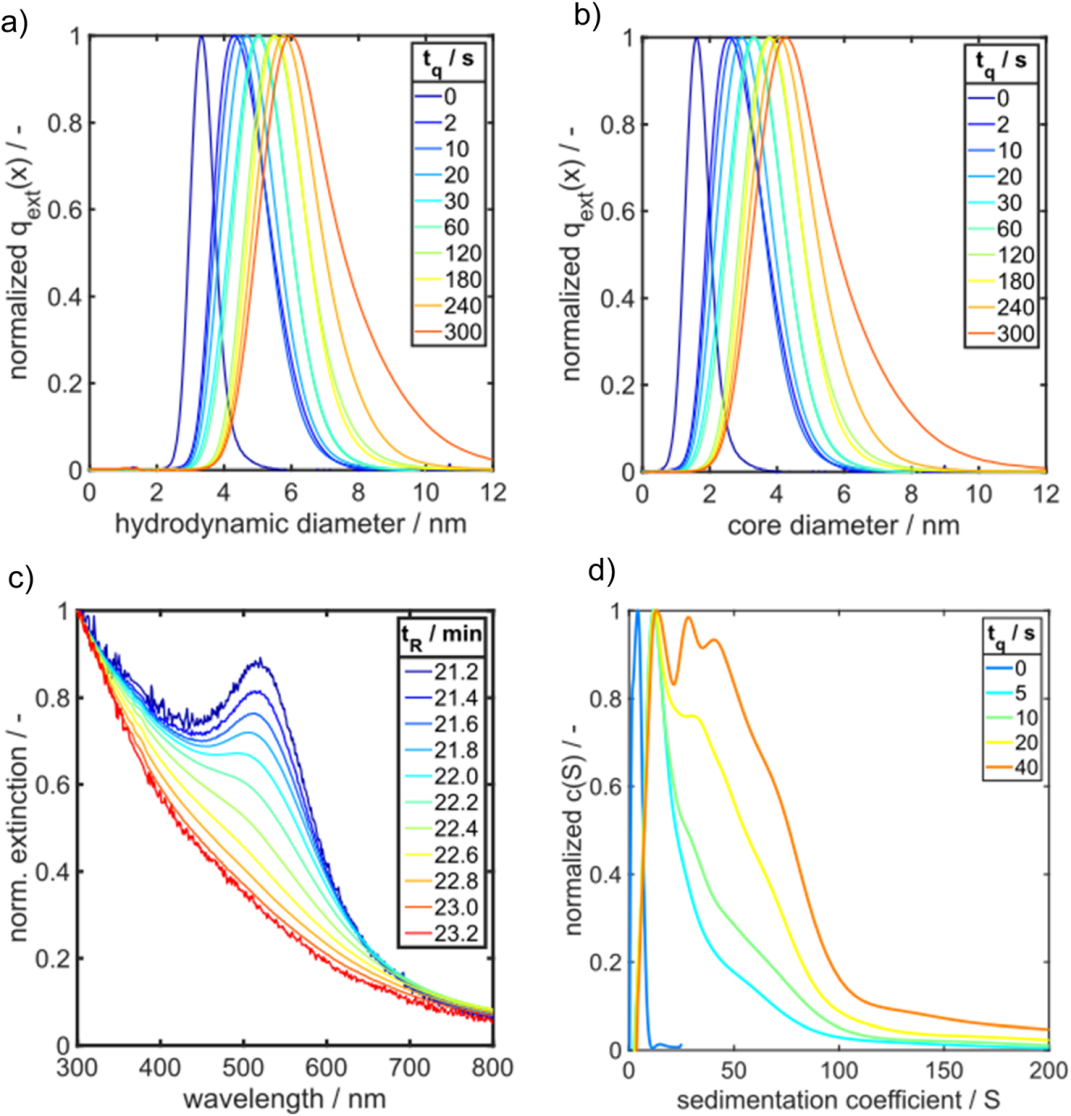
Comparison of HPLC-SEC and AUC results for GSH-Au NP samples. Normalized PSDs of quenched Au NP samples measured by HPLC-SEC are shown for (a) hydrodynamic diameter and (b) Au core diameter. (c) UV/vis spectra of different species in a GSH-Au NP sample quenched after 2 s as retrieved by HPLC-SEC. The components of the sample exhibit retention times *t*_R_ between 21 min and 23 min. (d) Sedimentation coefficient distributions of GSH-Au NP samples quenched between 0 s and 40 s measured by SV-AUC.

**Fig. 5:**
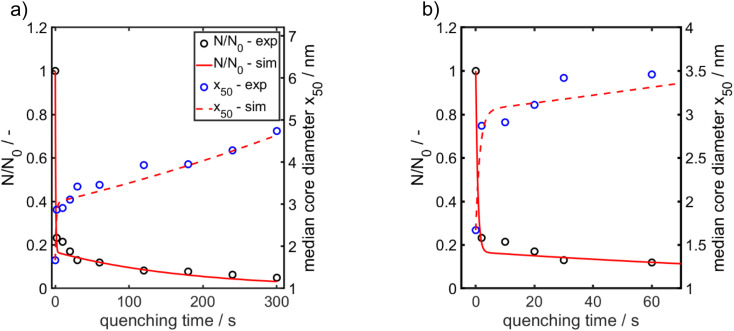
Experimentally obtained and modelled values for the temporal evolution of the median value for the Au NP core diameter (right axis) and of the relative Au NP number concentration (left axis). (a) Total time range. (b) Time range from 0–70 s enlarged for better visualization of the early stages. The particle number concentration at the beginning *N*_0_ amounts to 6.1 × 10^20^ m^−3^.

Fig. 4a presents extinction-weighted PSDs of the hydrodynamic diameter for samples quenched after *t*_q_ between 0 and 300 s. Fig. 4b shows the extinction-weighted PSDs of the Au NP core diameter as a function of *t*_q_. The core diameter was determined by subtracting twice the shell thickness of GSH (0.86 nm) from the hydrodynamic diameter. The density distributions of the Au core diameter shown in Fig. 4b were integrated to obtain the cumulative PSDs (see Fig. S4 in the ESI†). From the cumulative PSDs the median values of the core diameter were extracted and plotted *vs.* the quenching time (blue data points in Fig. 5). Initially (at 0 s), the PSD is quite narrow with a median core diameter of 1.6 nm, corresponding to Au_127_ clusters according to Negishi *et al.*^53^ The PSD of the sample quenched after 300 s exhibits a median value of the core diameter of 4.7 nm (Fig. 5). The Au NPs grow systematically in size with a maximum growth rate of about 0.50 nm s^−1^ at the beginning, taken from the slope of the particle size between the data points for *t*_q_ = 0 and 2 s in Fig. 5. For *t*_q_ > 2 s, the initial growth rate decreases continuously with increasing reaction time, see Fig. 5, and reaches finally (after 300 s) a value of 0.008 nm s^−1^ (taken from the slope between the two blue data points at *t*_q_ = 240 s and 300 s in Fig. 5a).

ESI-DMA-MS reveals that the sample “*t*_q_ = 0” consists of Au salt and Au_9_–Au_12_ clusters, whereat Au_10_ is the most frequently occurring cluster species, see Fig. S3 in the ESI.† This indicates that already during the initial mixing time, a part of the Au salt is reduced to the elemental state and Au clusters are formed. Thus, the rapid Au NP growth observed during the first 2 s must be caused by agglomeration of the clusters and/or integration of Au atoms or clusters in the forming Au NPs. In the ESI-DMA-MS spectra of the samples quenched at 2 s and 5 s, respectively, the signals of the Au salt and Au clusters are still present, but their intensity decreases with growing quenching time indicating that these species are consumed. The extinction at 400 nm E400 is directly proportional to the concentration of Au NPs and therefore allows to track the overall conversion of the Au salt to Au NPs quantitatively.^54,55^ According to Fig. S5 in the ESI,† E400 almost reaches its final value at a quenching time of 2 s, which is in line with the transition from rapid to slow Au NP growth. Thus, after 2 s the conversion of the Au salt to Au NPs has already reached a high level, so that further Au NP growth by integration of Au atoms or Au clusters should play only a minor role at *t*_q_ ≥ 2 s. As for quenching times ≥2 s the majority of Au is already integrated in the Au NPs, we anticipated that the further increase in size is driven mainly by agglomeration. According to the analysis of Lee for particle aggregation of lognormal PSDs, the temporal evolution of the mean particle size *x* obtained from the mean particle volume and the particle number concentration *N*, respectively, of a sample with lognormal PSD can be described by the eqn (3) and (4).^56^3
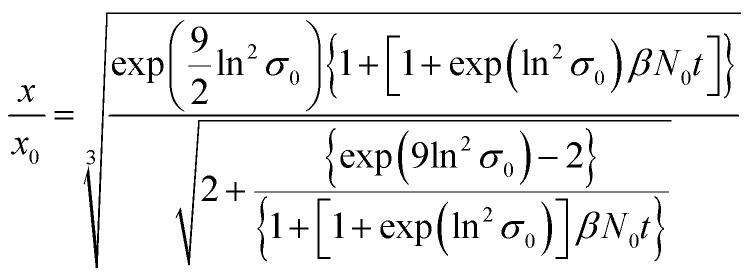
4
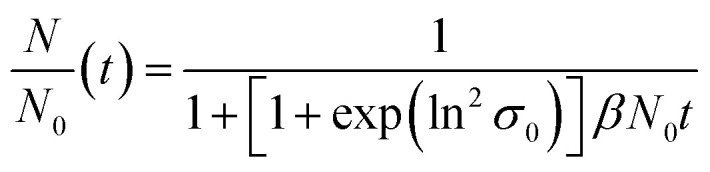


For *x* in eqn (3), we set the median values *x*_50,ext_ of the Au NP core diameter. The values for the volume of individual Au NPs were calculated from the core diameters by approximating the Au NPs as spheres, which is reasonable according to the STEM micrograph shown in Fig. 2b. The values for the particle number concentration *N* were determined from the measured lognormal PSDs, the volume of an individual particle of each size fraction and the total amount of Au (0.1 mol m^−3^). All the PSDs shown in Fig. 4b can be excellently described by lognormal distributions (see Fig. S6 in the ESI†) and the values for the geometric standard deviation *σ* were obtained by the lognormal fit (see Table S2 in the ESI†). The parameter *β* in eqn (3) and (4) is the agglomeration kernel for Brownian motion. *β* was determined from the experimental values of the particle number concentration by inverting eqn (4).^56^ The obtained values for *β* are shown in Fig. S7 in the ESI.† The values for *β* depend on the particle size and are in the range of 5 × 10^−23^ m^3^ s^−1^ and 1.3 × 10^−21^ m^3^ s^−1^ which is 4–6 orders of magnitude lower than the kernel for Brownian agglomeration in the absence of any colloidal stability (each particle collision step results in agglomeration). The latter is in the order of 10^−17^ m^3^ s^−1^.^56^ We set the PSD of the Au NP sample quenched after 0 s as starting point for the agglomeration process and obtained *x*_0_ = 1.67 nm, *σ*_0_ = 1.25 and *N*_0_ = 6.1 × 10^20^ particles per m^3^.

According to Fig. 5a and b, the experimentally obtained temporal evolutions of the Au NP core diameter (blue data points) and the relative Au NP number concentration *N*/*N*_0_ (black data points) are quite well represented by the agglomeration model (eqn (3) and (4), respectively, solid and dashed red lines).

In conclusion, agglomeration of the Au NPs must be the growth mechanism for reaction times ≥ the mixing time (*t*_q_ = 0 s). The geometric standard deviation *σ* of the sample quenched after 0 s is 1.25 and changes to 1.33 after 2 s. This latter value is surprisingly close to the value of 1.32 for a self-similar lognormal distribution and thus is a hint that already in the early stages particle growth is determined by aggregation of clusters.^57^ In the intermediate stages, the measured PSDs become a bit narrower, the standard deviations decrease by a maximum of 6% and approach again the value of the self-similar distribution after 300 s. The reason for this deviation might be additional growth of not fully converted molecular species.

To get more insight into the initial phase of the solid formation process, we analyse the extinction spectra at increasing retention times *t*_R_ for a sample obtained by addition of GSH 2 s after starting the reaction (Fig. 4c). From large to small retention times, we find that the extinction spectra evolve from an exponential decaying function to a typical spectrum of plasmonic Au NP. In SEC, the largest particles elute first followed by successively smaller fractions. The exponential decaying function give rise to Au clusters with semiconductor properties.^58^ Note that the number of gold atoms within the clusters cannot be determined from the spectra. Nevertheless, already at very short process times, the suspension consists of a mixture of Au clusters and plasmonic particles, as it can be seen in Fig. 4c for GSH-Au NP quenched at *t*_q_ = 2 s. For further comparison, the HPLC-SEC retention time *t*_R_ resolved UV/vis spectra for GSH-Au NP samples quenched after 0 s and 300 s, respectively, (*t*_q_ = 0 s and *t*_q_ = 300 s) are shown in Fig. S8 in the ESI.† As demonstrated by Fig. S8a,† the sample *t*_q_ = 0 s is containing a mixture of Au clusters shown by the exponential decaying UV/vis spectrum for each *t*_R_ but no plasmonic Au NPs are detected. In contrast, the GSH-Au NPs quenched at *t*_q_ = 300 s display only characteristic spectra of plasmonic Au NPs with increasing *t*_R_ (see Fig. S8b in the ESI†).

**Fig. 6:**
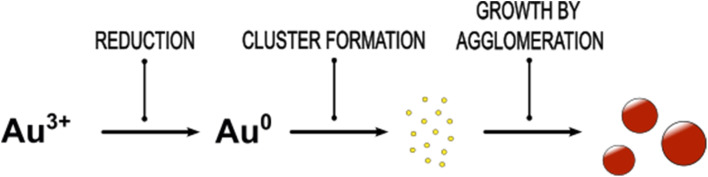
Proposed formation mechanism of small spherical plasmonic Au NPs by fast reduction of Au(iii) ions by the strong reducing agent NaBH_4_. As first semiconducting Au clusters are formed due to supersaturation of Au atoms. The Au clusters grow by agglomeration to plasmonic Au NPs.

Obviously, the synthesis of Au NPs starts with the formation of small semiconducting Au species, which grow by agglomeration to a certain size, see growth scheme in Fig. 6. This is in line with the findings of Polte *et al.*^21^ However, the scattering experiments performed by Polte *et al.* did not allow to distinguish between the semiconducting and metallic behaviour of the Au NP. Our data suggest in addition that the Au NP formation dynamics starts with the formation of non-plasmonic Au clusters, continues by the formation of intermediates and ends up with plasmonic (metallic) Au NPs. In particular, the quenching of the reaction progress allows to extract information on PSDs and optical properties of the intermediates at different reaction times without requirement of methods with limited availability like SAXS/SANS.

By comparing the PSDs derived from HPLC-SEC with the sedimentation coefficient distributions of GSH-Au NP measured by AUC (Fig. 4d), the same overall trend is shown for a time range of 0 s to 40 s. Due to the better resolution of the AUC measurement intermediates are visible more clearly. A very narrow sedimentation coefficient distribution with one main species and two smaller shoulders results from the GSH-Au NP sample quenched at 0 s. By following the GSH-Au NP samples over time, the sedimentation coefficient distribution (SCD) shows still one main species shifting further to higher sedimentation coefficients, while the distribution itself is broadening and showing evolving shoulders. This indicates that very small and narrowly distributed species are present at the beginning. These species are vanishing over time while bigger intermediates and Au NPs are forming. In accordance with the results from HPLC-SEC (Fig. 4c), the AUC measurements also provide evidence that the samples quenched after short times consist of a mixture of small Au clusters and plasmonic Au NPs.

### Continuous synthesis

Due to the fast Au NP formation, a mixing dependency is expected. As in a batch synthesis mixing is hard to control, we conduct the Au NP synthesis in a classical continuous T-mixer setup (Fig. 1a). The flow and mixing conditions in a T-mixer are well-known for inflow volume ratios of 1 : 1, as it has been used beforehand for the discussed batch synthesis.^59–62^

**Fig. 7:**
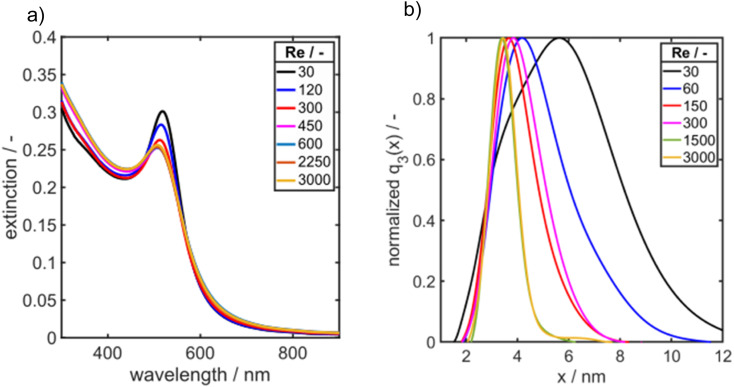
(a) UV/vis spectra and (b) PSDs *q*_3_(*x*) of Au NPs synthesized in a T-mixer at increasing Reynolds number as measured by AUC.

The educt solutions are transported through a T-mixer by a syringe pump, see scheme in Fig. 1a. To systematically investigate the influence of mixing of the educt solutions on the final PSDs of the Au NPs, the dimensionless inflow velocity (Reynolds number, eqn (1)) of the synthesis was increased successively from 30 to 3000 which corresponds to flow rates from 0.71 to 70.8 mL min^−1^. In Fig. 7a, the UV/vis spectra of the synthesized Au NPs are shown as a function of Re number.

**Fig. 8:**
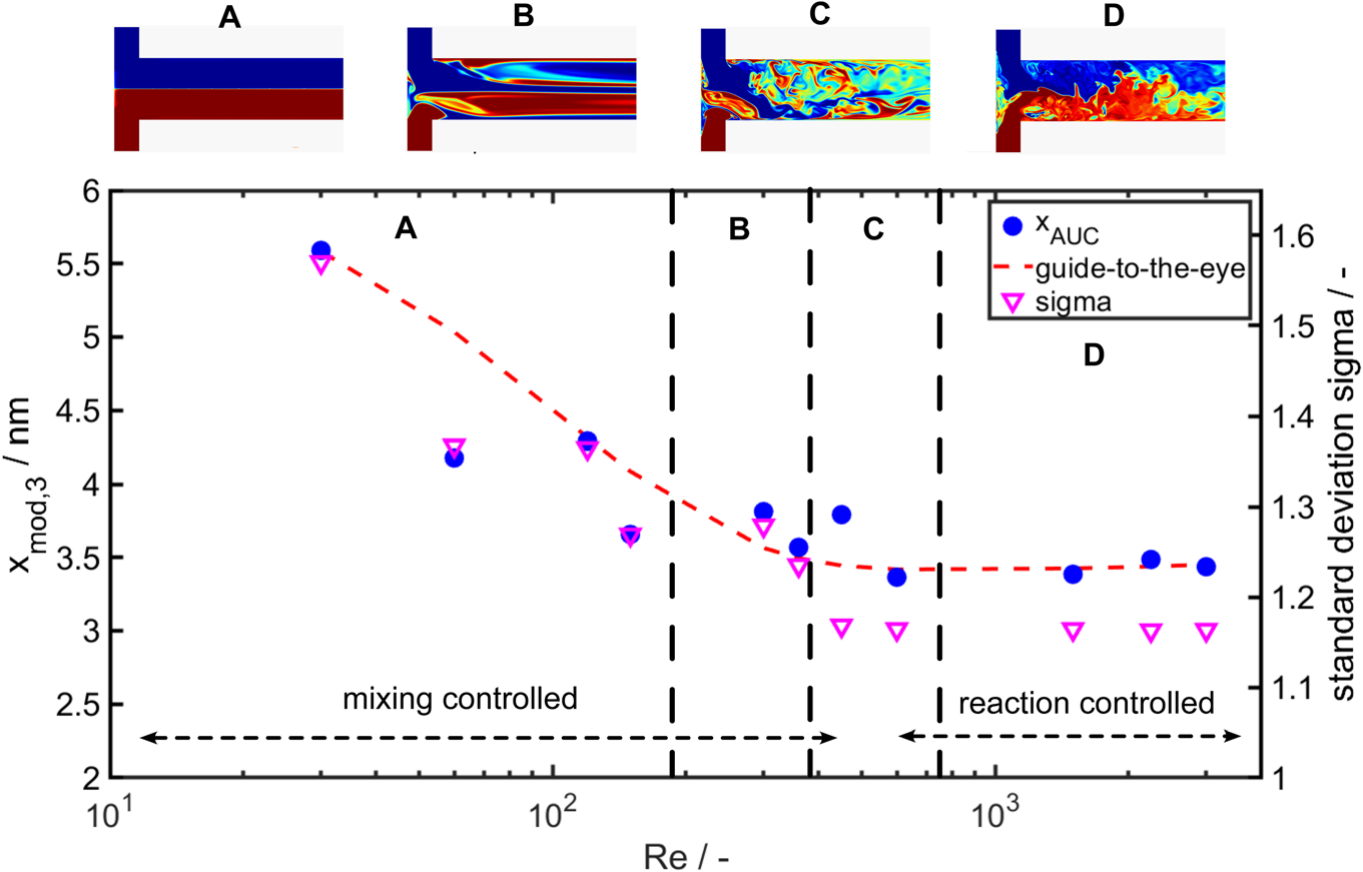
Modal particle size *x*_mod,3_ of the *q*_3_(*x*) measured by AUC over the respective Reynolds number (Re) (left axis) and standard deviation of the lognormal PSDs (right axis).

As it can be seen the extinction at the LSPR is decreasing from Re 30 to Re 600 and is staying constant for further increasing Re (Fig. 7a). Fig. 7b shows the corresponding PSDs over increasing Re. With increasing Reynolds number the modal value (*x*_mod,3_) and the width of the distribution *σ* is decreasing as it can be seen in Fig. 7b and 8.

While the largest values for the particle size (5.8 nm) and *σ* (1.5) are measured for Re 30, the smallest particle size (3.4 nm) and standard deviation *σ* (1.16) are obtained for Re 600. For Re > 600 the particle size and the geometric standard deviation *σ* stay almost constant.

In the investigated Re range the particle size decreases by more than 50% and the geometric standard deviation decreases by 26%. This change in particle size and PSD width due to the increase of energy input shows a clear mixing dependency as it is expected for such a fast reaction and is in good agreement to the literature.^60–62^ Remarkably, an increase of the energy input, which overall improves the mixing rate, results not only in a decrease of the modal particle size but also the PSD becomes narrower (see Fig. 7b and 8 right axis). Thus, it appears that the general sequence of faster mixing inducing faster supersaturation build-up, which favours nucleation over molecular growth, results in smaller particles and applies here. While overall the mean particle size decreases with the Reynolds number, we observe notable fluctuations in the Reynolds number regime from Re = 350 to Re = 700.

To get insights into the local mixing behaviour of the T-mixer used in the experiments, we performed direct numerical simulations of the mixing process (for details on the numerical approach see^59^). The obtained flow patterns from the simulations in Fig. 8 provide information about the mixing rate and homogeneity at the investigated Reynolds numbers. The change in the large-scale flow structures induced by different fluid instabilities at Re ∼300, 510 and 750 affects the mixing homogeneity (see Fig. 8) and leads to increased, yet overall small, scatter of disperse properties. Indeed, a more uniform mixing behaviour (larger Re number) correlates well with a narrower PSD of the Au NPs obtained at larger Re.

## Conclusion

We have shown that the fast Au NP formation process due to reduction of Au(iii) ions by sodium borohydride can be frozen by adding GSH as quencher. In fact, quenching the fast particle formation by highly efficient stabilizers allows to employ the full plethora of particle characterization techniques. The analysis of the quenched samples by the precise *ex situ* analytics HPLC-SEC, ESI-DMA-MS and AUC enables the quantitative determination of the growth kinetics and the detection of intermediate species. We show that already during the initial mixing time semiconducting Au clusters with Au_10_ as main species are formed which grow rapidly to Au NPs within 2 s. After 2 s the growth regime turns from rapid to slow because the majority of the Au precursor is now consumed. The initial fast growth as well as the slow growth regime can be reasonably described by an agglomeration model indicating that agglomeration is the main growth mechanism. While the Au NP growth in the slow regime proceeds, Au clusters are increasingly converted to plasmonic Au NPs. From the initially fast growth rate we conclude that mixing plays major role for the final PSD. As mixing is hard to control in batch, we established a continuous synthesis using a T-mixer with well-known and defined mixing behaviour. We found that the average size and the width of the PSDs decrease with increasing Re because of better mixing.

The introduced quenching method is not limited to Au clusters or spherical Au NPs. It can be extended to monitor the formation of non-spherical particle shapes or of other nanoparticle systems. While the growth of spherical or triangular silver nanoparticles can also be quenched by GSH, the transfer to other systems such as Pt catalyst particles might require adaptation of the structure of the quencher molecule. Systematic exploration of highly efficient stabilizing molecules may open many novel pathways for the in-depth exploration of growth mechanisms and growth kinetics of functional nanoparticle systems.

## Conflicts of interest

There are no conflicts to declare.

## Supplementary Material

RA-013-D3RA02195E-s001
