# Supplementary material for: Efficient quenching sheds light on early stages of gold nanoparticle formation
Source: RSC Adv. 2023 Jun 14;13(26):18001–13. doi: 10.1039/d3ra02195e (PMC10265400; doi:10.1039/d3ra02195e)
Supplement: RA-013-D3RA02195E-s001 [file RA-013-D3RA02195E-s001.pdf]

Electronic Supplementary Material (ESI) for RSC Advances.
This journal is © The Royal Society of Chemistry 2023

Supporting Information

Efficient quenching sheds light on early stages of gold nanoparticle formation

Markus Biegel, Tobias Schikarski, Paola Cardenas Lopez, Lukas Gromotka, Christian Lübbert, Andreas Völkl, Cornelia Damm, Johannes Walter, Wolfgang Peukert*

Institute of Particle Technology (LFG), Friedrich-Alexander-Universität Erlangen-Nürnberg (FAU), Cauerstrasse 4, 91058 Erlangen, Germany; Interdisciplinary Center for Functional Particles Systems (FPS), Friedrich-Alexander-Universität Erlangen-Nürnberg (FAU), Haberstraße 9a, 91058 Erlangen, Germany.
* Corresponding author: E-mail: wolfgang.peukert@fau.de

As mentioned in the main text, the shell thickness of GSH was retrieved via SV-AUC experiments followed by combined sedimentation-diffusion analysis of a $Au_{10}(GSH)_{10}$ sample, see Figure S1. As described in the literature, thiolate-protected Au clusters form with distinct numbers of Au and thiol ligand atoms[1]. In particular for Au_{10} clusters, the sulphur present in the GSH molecule will bind to each Au atom, resulting in the formation of defined $Au_{10}(GSH)_{10}$ species. For the Sedfit analysis, the unknown partial specific volume was adjusted to equal the known molar weight of $Au_{10}(GSH)_{10}$ of 5.03 kDa. We obtained a partial specific volume of 0.372 cm^3/g. The diffusion coefficient of $1.79x10^{-10}$ m^2/s is retrieved from the Svedberg equation and a hydrodynamic diameter of 2.4 nm is obtained via the Stokes-Einstein equation. Furthermore, by considering a spherical shape of the core and the bulk density of Au, the diameter of Au_{10} is calculated to be 0.68 nm. We proceed by simply extracting the core diameter from the hydrodynamic one, yielding a shell thickness of 0.86 nm.

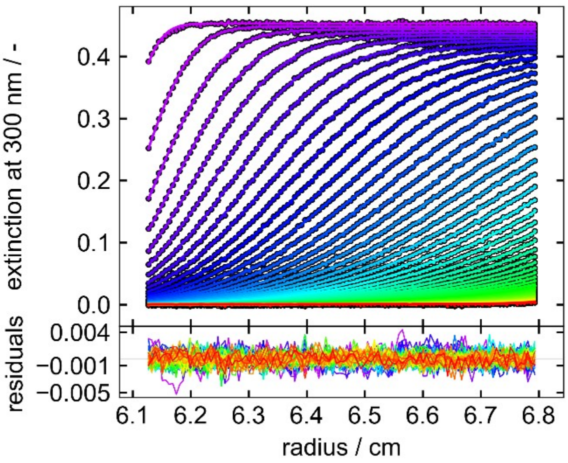

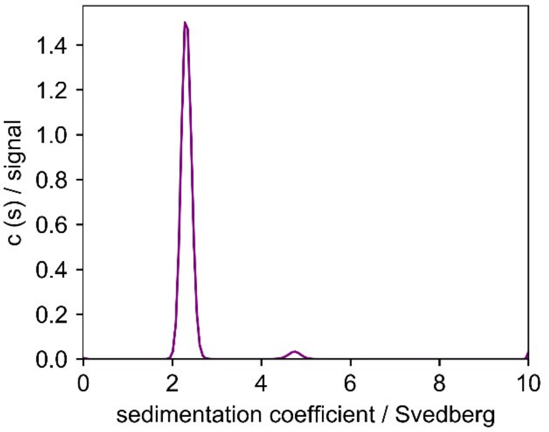

Figure S1: a) Sedimentation velocity data of $Au_{10}(GSH)_{10}$ (dots) together with best-fit data from the continuous species c(s) model (lines) and resulting random residuals. b) Sedimentation coefficient distribution of $Au_{10}(GSH)_{10}$ obtained by c(s) analysis.

In order to determine the PSDs of the quenched GSH-Au NPs by SEC, 20 µl of the Au NP standard dispersions and the Au nanocluster sample were injected sequentially into the chromatograph. Details on the synthesis of the Au nanocluster sample can be found in literature[2]. The normalized chromatograms are shown in Figure S2a. From the volume mean NP sizes (see Table S1) and the respective retention volumes, the calibration curve (Figure S2b) was then determined. For the evaluation of the data points in the calibration curve, a 3rd order polynomial fit (R^2 = 0.995) was used. The size corresponding to the measured retention time of 23.3 min, was taken from Soleilhac et al.[3] assuming that the sample mainly consists of $Au_{15}GSH_{13}$. Hence, the hydrodynamic diameter according to Soleilhac et al. is 2.9 nm.

From the chromatograms and the calibration curve in Figure S2, a clear trend towards larger retention volumes with decreasing NP size is observed. Thus, the retention is dominated by a size-exclusion (SEC) mechanism and the PSDs of the quenched GSH-Au NPs can be determined from the fitted calibration curve.

Table S1: Volume mean NP sizes and standard deviations σ of Au standard dispersions determined by DLS.

	5 nm	10 nm	20 nm	30 nm	40 nm	50 nm	60 nm	80 nm
$x_{3,mean}$ / nm	8.4	13.4	22.0	32.2	40.4	50.9	63.2	79.5
σ / nm	0.5	1.6	0.2	0.5	0.7	1.3	2.1	2.2

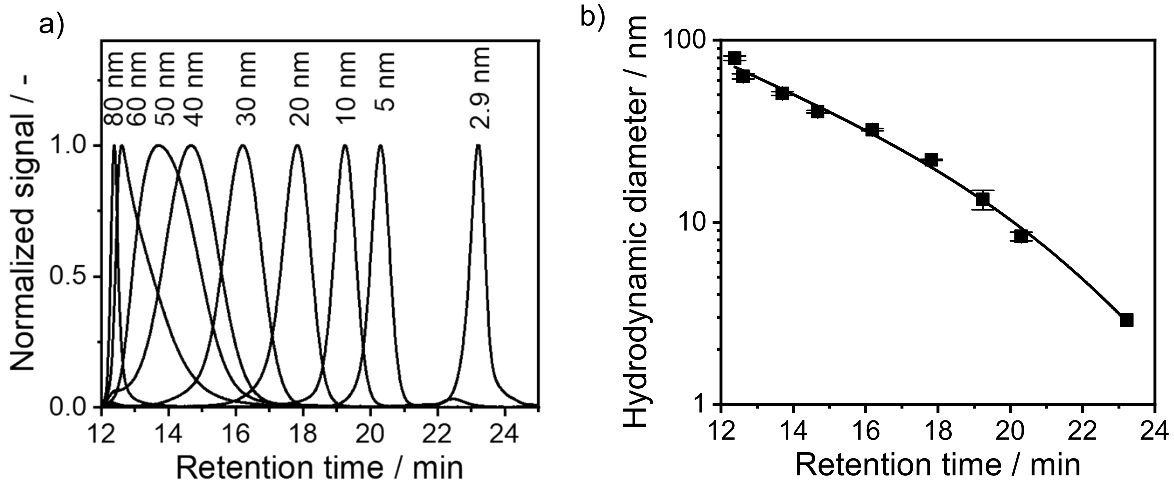

Figure S2: Normalized chromatograms of Au standard dispersions (a). SEC calibration curve for the determination of PSDs. The symbols indicate the individual calibration points and the solid line the 3rd order polynomial fit. The calibration curve is constructed from the volume mean diameter of Au standard dispersions measured by DLS and the respective retention times except the point of 2.9 nm which was compared with the results of Soleilhac et al.[3] (b).

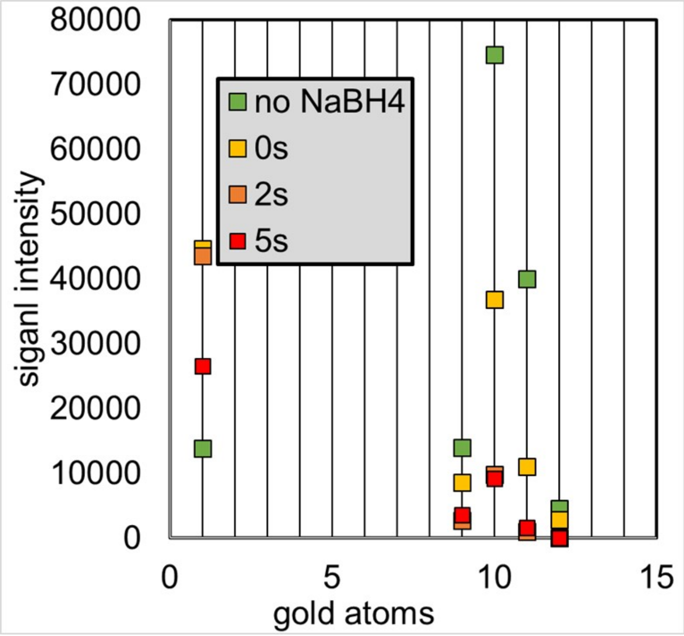

Figure S3: ESI-DMA-MS signal intensity for different Au species as a function of quenching time t_q.

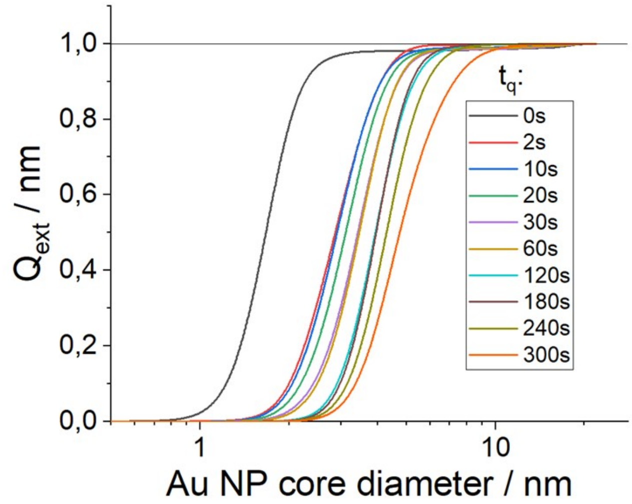

Figure S4: Extinction weighted cumulative distribution of the Au NP core diameter as a function of the quenching time t_q.

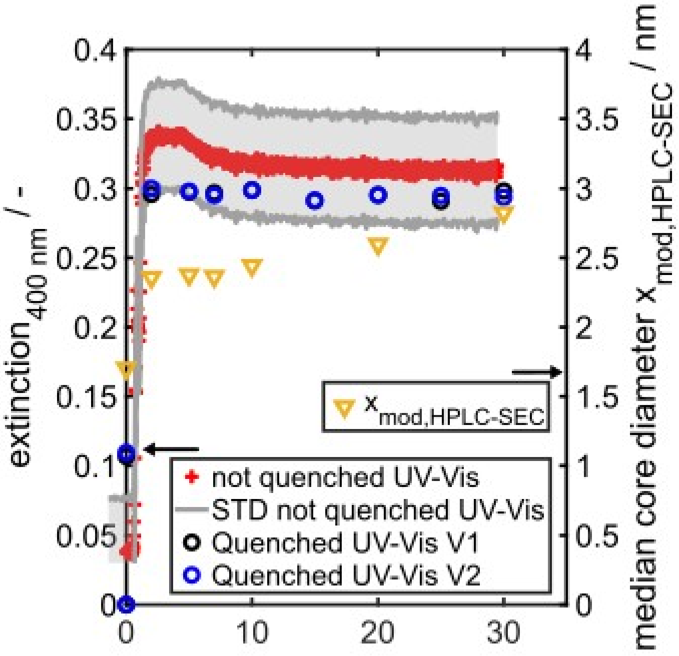

Figure S5: Time evolution of the extinction at 400 nm E400 (left axis) and time resolved median core diameter from number density PSD of HPLC-SEC (right axis). The black and the blue data points marked with "V1" and "V2" in the legend are the values of two independent quenching experiments to demonstrate the high reproducibility of the quenching method.

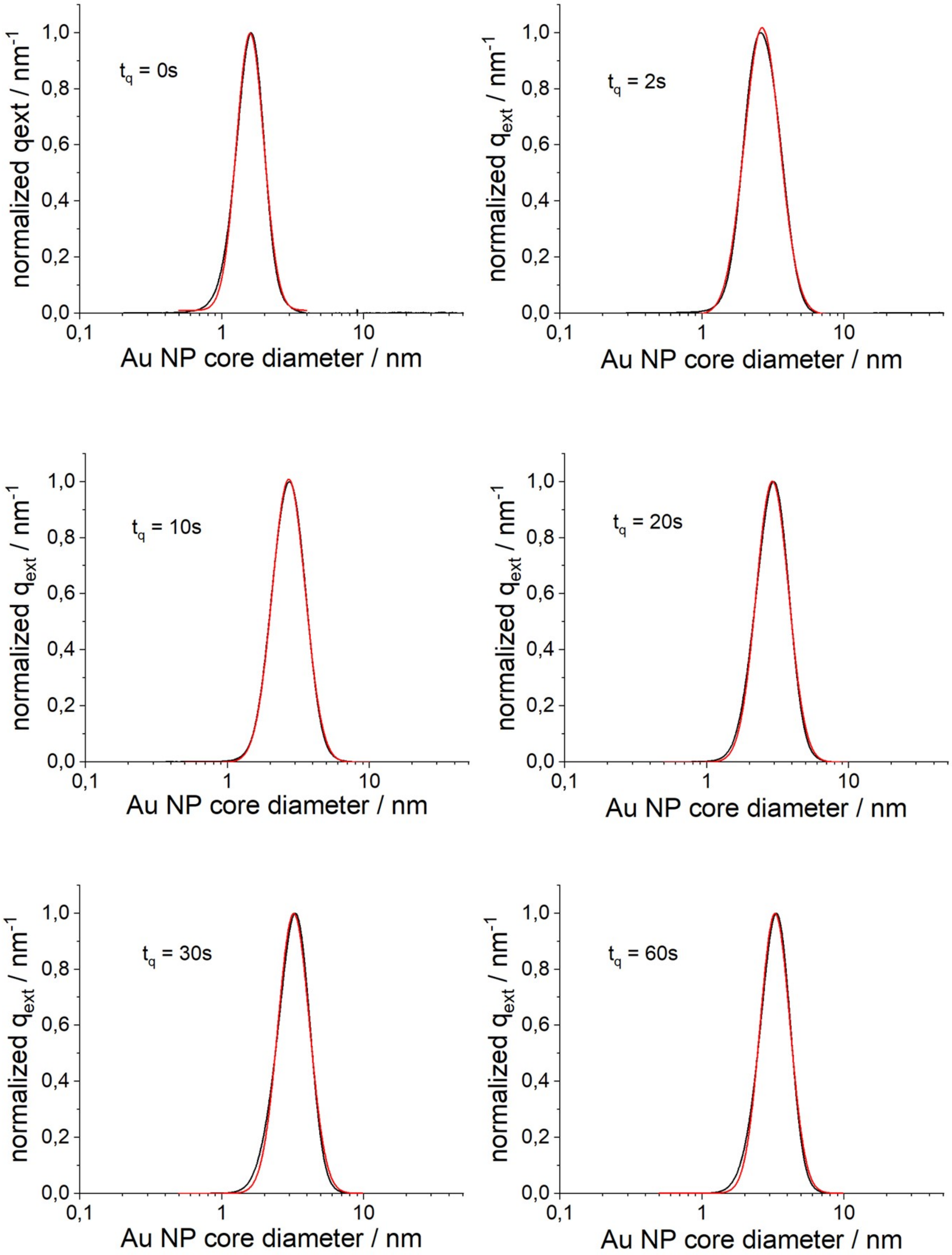
t_q = 0s
t_q = 2s
t_q = 10s
t_q = 20s
t_q = 30s
t_q = 60s
normalized qext / nm-1
normalized q_ext / nm-1
Au NP core diameter / nm
0,0
0,2
0,4
0,6
0,8
1,0
0,1
1
10

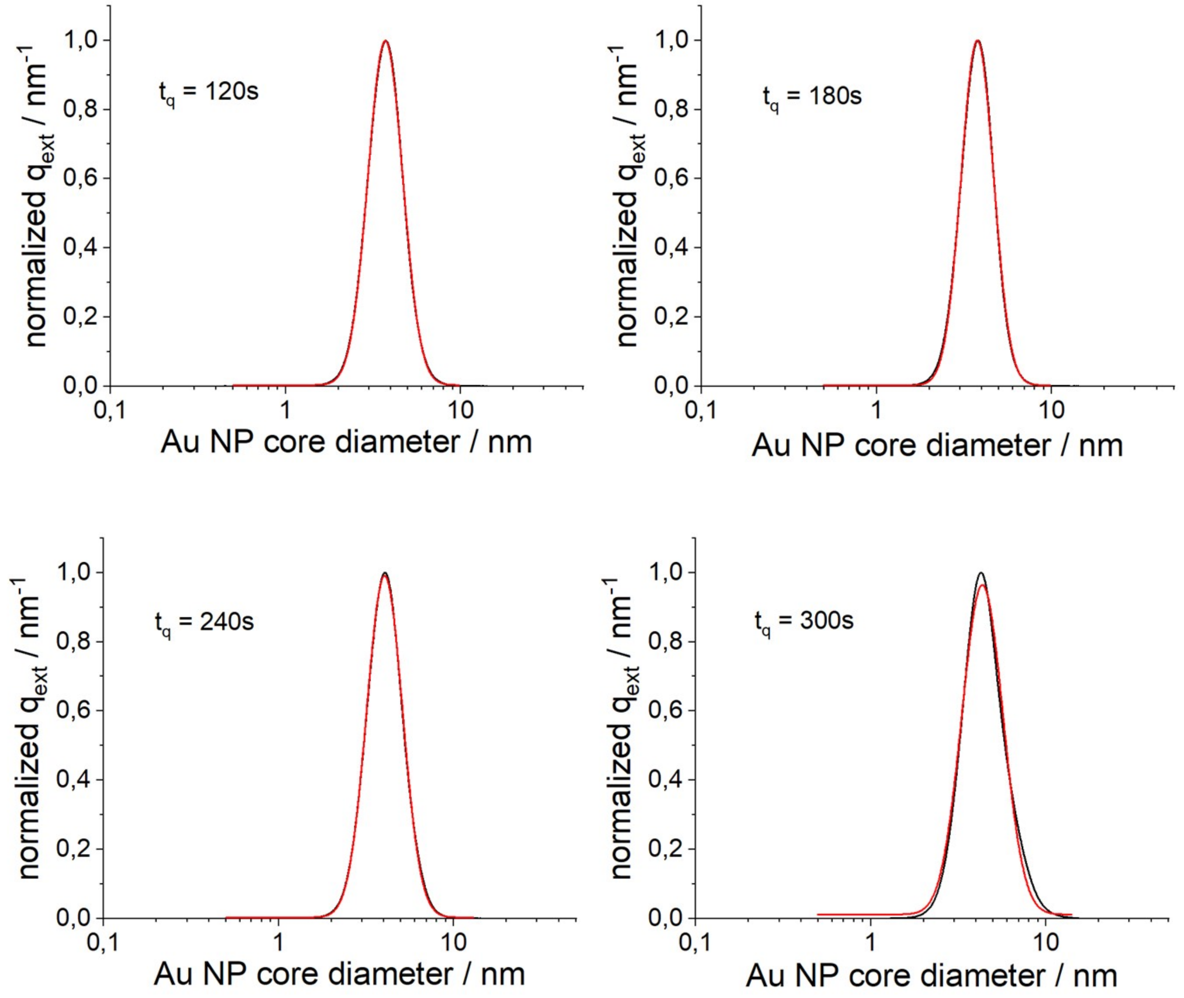

Figure S6: Lognormal fits (red curves) of the Au NP core diameter distributions obtained from HPLC-SEC (black curves).

Table S2: Parameters of the lognormal fits shown in Figure S6.

**Quenching time t_q / s**	**x_c / nm**	**w=ln(σ)**	**σ**	**R^2**
0	1.66±0.01	0.2223±0.0006	1.25±0.01	0.9976
2	2.87±0.01	0.2886±0.0004	1.33±0.01	0.9986
10	2.90±0.01	0.2688±0.0002	1.31±0.01	0.9996
20	3.11±0.01	0.2636±0.0004	1.30±0.01	0.9982
30	3.43±0.01	0.2550±0.0006	1.29±0.01	0.9960
60	3.46±0.01	0.2416±0.0005	1.27±0.01	0.9970
120	3.91±0.01	0.2288±0.0001	1.26±0.01	0.9998
180	3.94±0.01	0.2133±0.0002	1.24±0.01	0.9996
240	4.26±0.01	0.2358±0.0001	1.27±0.01	0.9997
300	4.66±0.01	0.2701±0.0007	1.31±0.01	0.9938

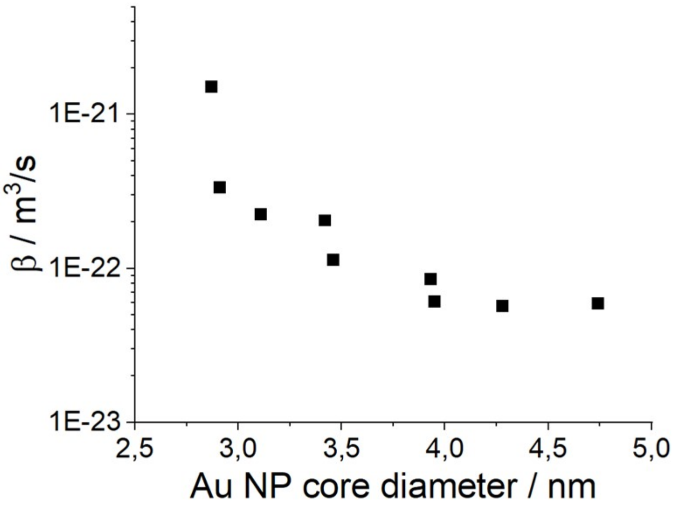

Figure S7: Agglomeration kernel for Brownian motion used in equations (3) and (4) as a function of particle size.

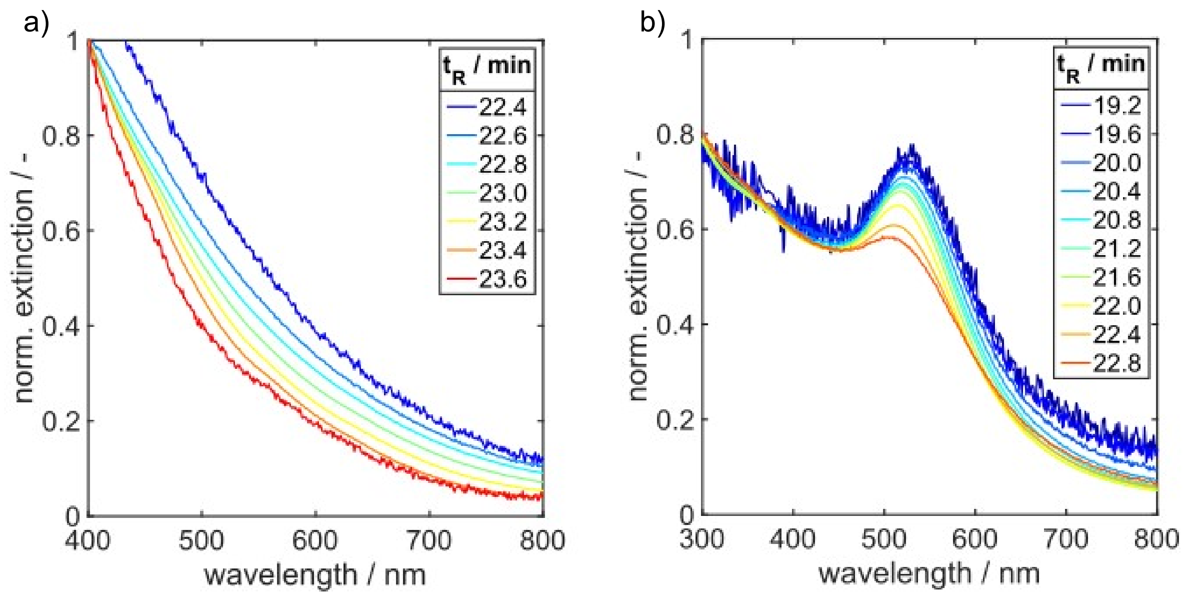

Figure S8: Retention time t_R resolved UV/Vis spectra extracted from the chromatogram of a) A GSH-Au NP sample quenched at t_q = 0 s and b) A GSH-Au NP sample quenched at t_q = 300 s.

References

1 Y. Negishi, K. Nobusada and T. Tsukuda, Glutathione-protected gold clusters revisited: bridging the gap between gold(I)-thiolate complexes and thiolate-protected gold nanocrystals, *J. Am. Chem. Soc.*, 2005, **127**, 5261–5270.

2 Y. Niihori, D. Shima, K. Yoshida, K. Hamada, L. V. Nair, S. Hossain, W. Kurashige and Y. Negishi, High-performance liquid chromatography mass spectrometry of gold and alloy clusters protected by hydrophilic thiolates, *Nanoscale*, 2018, **10**, 1641–1649.

3 A. Soleilhac, F. Bertorelle, C. Comby-Zerbino, F. Chirot, N. Calin, P. Dugourd and R. Antoine, Size Characterization of Glutathione-Protected Gold Nanoclusters in the Solid, Liquid and Gas Phases, *J. Phys. Chem. C*, 2017, **121**, 27733–27740.
